# Early Cardiac Biomarkers for the Detection of Cancer Therapy-Related Cardiac Dysfunction in Breast Cancer: A Systematic Review and Meta-Analysis

**DOI:** 10.3390/jcm15207882

**Published:** 2026-10-13

**Authors:** Nazym Askambayeva, Zhuldyz Myrzabay, Ahmad J. Harahsheh, Reezal Ishak, Aigerim Biniyazova, Mohamad Aljofan

**Affiliations:** 1 Department of Biomedical Sciences, School of Medicine, Nazarbayev University, 5/1 Kerey and Zhanibek Khandar str., Astana 010000, Kazakhstan; nazym.askambayeva@nu.edu.kz (N.A.); zhuldyz.myrzabay@nu.edu.kz (Z.M.); aigerim.biniyazova@nu.edu.kz (A.B.); 2 Department of Biology and Biotechnology, Faculty of Science, The Hashemite University, Zarqa 13133, Jordan; ahmad_harahsheh@hu.edu.jo; 3 Institute of Medical Science Technology [UniKL MESTECH], Universiti Kuala Lumpur, A1-1, Jalan TKS 1, Taman Kajang Sentral, Kajang 43000, Selangor, Malaysia; reezal@unikl.edu.my; 4 National Laboratory Astana, Center of Life Sciences, Nazarbayev University, Astana 010000, Kazakhstan

**Keywords:** cardiotoxicity, cardiac biomarkers, troponin, NT-proBNP, anthracyclines, biomarkers

## Abstract

**Background/Objectives:** Chemotherapy-induced cardiotoxicity is a serious complication in breast cancer treatment, particularly with anthracyclines and HER2-targeted therapy. To evaluate the association between early elevations of cardiac markers (troponin and NT-proBNP) and the development of chemotherapy-induced cardiotoxicity in adult patients with breast cancer. **Methods:** This systematic review and meta-analysis were conducted according to PRISMA guidelines and registered with PROSPERO (CRD420261301170). A systematic search of PubMed/MEDLINE, Cochrane CENTRAL, and Web of Science databases was conducted. Studies evaluating cardiac markers in breast cancer patients receiving chemotherapy were included in the review. **Results:** Fourteen studies were included in the qualitative synthesis. Five studies were included in the meta-analysis. Elevated troponin levels were associated with higher odds of subsequent cardiac dysfunction; however, the pooled association was not statistically significant [OR = 3.40, 95% CI 0.69–16.67, *p* = 0.13] and showed substantial heterogeneity [I^2^ = 86.97%]. A leave-one-out sensitivity analysis was used to identify a study that exerted a substantial influence; its exclusion eliminated between-group heterogeneity and attenuated the pooled effect estimate. **Conclusions:** Early troponin elevation may provide a biological signal of myocardial injury during cancer therapy. However, the association between elevated troponin level and cancer therapy-related cardiac dysfunction did not reach the threshold of statistical significance and showed substantial heterogeneity. Troponin and NT-proBNP may provide complementary information to cardiac imaging, but their predictive performance and clinical utility require further prospective validation.

## 1. Introduction

Breast cancer is the most frequently diagnosed cancer worldwide today. It remains a leading cause of morbidity and mortality among women [1]. Advances in systemic therapy have significantly improved treatment outcomes for both early and advanced stages of breast cancer. Furthermore, detailed studies of molecular subtypes of breast cancer have improved not only our understanding but also the precision of treatment [2]. These treatments include anthracycline-based chemotherapy and HER2-targeted therapy using trastuzumab [3].

Cancer therapy can negatively impact cardiac structure and/or function, manifesting as asymptomatic cardiac dysfunction or symptomatic heart failure. These changes are collectively referred to as cancer therapy-associated cardiotoxicity [CTRCD] [4]. The authors identify five categories—(i) cardiac dysfunction: cardiomyopathy/heart failure [HF], (ii) myocarditis, (iii) vascular toxicity, (iv) hypertension, (v) arrhythmias and QTc prolongation—which define the most common adverse cardiovascular events during modern cancer therapy. The 2022 European Society of Cardiology (ESC) Cardio-Oncology Guidelines highlight that cardiovascular toxicity is a major challenge in modern cancer care and recommend structured surveillance for patients receiving treatment with potentially cardiotoxic agents, including anthracyclines and anti-HER2 therapy [5]. The use of chemotherapeutic agents in the treatment of breast cancer is limited due to the development of cardiotoxicity, and long-term cardiotoxicity can lead to death in approximately one-third of patients. Doxorubicin and anthracyclines are significant causes of acute and chronic cardiotoxicity in cancer patients [6,7]. These drugs belong to the class of anthracycline antibiotics, cytotoxic agents that entered clinical practice in the 1960s [8].

Hypokinetic cardiomyopathy, a manifestation of anthracycline-induced cardiotoxicity, was first described in 1967 [9]. The risk of anthracycline-induced cardiotoxicity increases with the use of other drugs that can increase its incidence. Trastuzumab, although highly effective in the treatment of breast cancer, disrupts myocyte survival pathways [10,11,12]. The biological mechanisms underlying CTRCD are complex and vary depending on the drug, but oxidative stress, mitochondrial damage, and cardiomyocyte death play a central role. Anthracyclines are known to induce the formation of reactive oxygen species and direct myocardial injury, whereas trastuzumab-associated toxicity is more closely linked to disruption of cardiomyocyte survival signaling pathways. These mechanisms help explain why myocardial injury may begin before the development of clinically evident cardiac dysfunction [13].

Currently, cardiotoxicity monitoring relies primarily on echocardiographic assessment of left ventricular ejection fraction (LVEF). This is most defined as a decrease in LVEF by ≥5% in symptomatic patients [or ≥10% in asymptomatic patients] from baseline to an LVEF < 55% [14]. However, changes detected by imaging techniques often become apparent only after cardiac injury has already occurred, which may limit the potential for timely cardioprotective intervention [15]. Reviews of myocardial strain analysis in cardio-oncology also note that traditional LVEF-based monitoring may underestimate early myocardial dysfunction [16]. For this reason, cardiac markers are increasingly gaining attention as tools for the early detection, assessment, and monitoring of myocardial injury caused by CTRCD. Cardiac troponins reflect cardiomyocyte damage, while natriuretic peptides such as BNP and NT-proBNP reflect myocardial wall stress and hemodynamic load [17,18]. In contrast, changes in global longitudinal strain (GLS) and LVEF provide evidence of evolving functional cardiac impairment. Therefore, biomarker elevation should not itself be considered synonymous with clinically meaningful CTRCD. A study by Cardinale et al. (2000) [19] demonstrated that troponin I (TnI), a sensitive and specific marker of myocardial injury in adults receiving high-dose chemotherapy, can predict its severity at a very early stage and the development of future left ventricular dysfunction. Previous studies have shown that elevated biomarker levels during chemotherapy can identify patients at increased risk of subsequent ventricular dysfunction and may facilitate earlier initiation of cardioprotective strategies [20].

However, the clinical utility of these biomarkers in patients with breast cancer remains uncertain. Existing studies vary significantly in chemotherapy regimens, patient characteristics, timing of biomarker measurement, assay sensitivity, cutoff values, and definitions of cardiotoxicity. Consequently, the available data are heterogeneous, and consensus on the optimal use of troponin and NT-proBNP in routine clinical monitoring has not been reached [21].

While current guidelines from the European Society of Cardiology support the use of cardiac biomarkers for risk stratification, they are largely based on heterogeneous data and expert consensus rather than robust pooled quantitative data.

Therefore, this systematic review and meta-analysis aimed to evaluate the association between early elevations of cardiac biomarkers, including troponin and NT-proBNP, and subsequent CTRCD in adult patients with breast cancer, and to assess the current evidence on their potential prognostic role and clinical applicability.

## 2. Materials and Methods

### 2.1. Study Design

This systematic review and meta-analysis were carried out following Preferred Reporting Items for Systematic Reviews and Meta-Analyses (PRISMA) guidelines (Supplemental Table S1). This study aimed to evaluate the link between early cardiac marker elevations and chemotherapy-induced cardiotoxicity in adult breast cancer patients. The study was prospectively registered in the PROSPERO database on 4 February 2026, with the number CRD420261301170. The information provided during registration has not been changed.

### 2.2. Search Strategy

A comprehensive literature search was conducted in PubMed/MEDLINE, Cochrane CENTRAL, and Web of Science. A more detailed version of the search strategy is presented in Supplemental Table S2.

For the qualitative synthesis (systematic review), only studies published between 2020 and 2025 were included to reflect current clinical practice, including the use of highly sensitive cardiac marker assays and modern chemotherapy regimens.

For the quantitative synthesis (meta-analysis), relevant studies published outside this period were additionally included if they provided sufficient data for pooling. This approach was adopted to increase statistical power and include earlier clinical data on the association between cardiac markers and chemotherapy-induced cardiotoxicity, particularly from basic research conducted before the widespread use of highly sensitive assays. The search strategy aimed to identify studies assessing the role of early cardiac markers in detecting chemotherapy-induced cardiotoxicity in patients with breast cancer. A predefined search strategy was developed using a combination of Medical Subject Headings (MeSH) and free-text terms, including “breast cancer,” “chemotherapy-induced cardiotoxicity,” and “cardiac biomarkers” (e.g., troponins, natriuretic peptides), as well as terms related to early diagnosis.

Only studies published in English were included. The complete electronic search strategy for all databases is presented in Supplementary Table S2.

### 2.3. Eligibility Criteria

Studies were included in this systematic review and meta-analysis if they met the following inclusion criteria:

Population: This review included studies involving adult (≥18 years) patients with non-metastatic (stages I–III) breast cancer who received potentially cardiotoxic chemotherapy, including anthracyclines and/or HER2-targeted therapy. The analysis was limited to patients with breast cancer to reduce clinical heterogeneity and improve comparability across studies, as treatment regimens, cumulative drug doses, and cardiotoxicity risk profiles are more consistent in this population.

Exposure/Predictors: Assessment of circulating cardiac biomarkers, including high-sensitivity cardiac troponin (hs-cTnI or hs-cTnT) and natriuretic peptides (BNP, NT-proBNP), before, during, or shortly after treatment.

Comparator: Patients with normal versus elevated biomarker levels, or baseline versus post-treatment [follow-up] measurements.

Outcomes: Occurrence of chemotherapy-induced cardiotoxicity, defined as a decline in LVEF and/or development of clinical heart failure, or other cardiac events as defined by the study authors.

Prospective or retrospective observational studies (cohort, case–control) and randomized controlled trials.

The following exclusion criteria were applied:

Pediatric populations (<18 years).

Studies involving non-chemotherapeutic cardiotoxic treatments only (e.g., chest radiation without systemic therapy).

Non-original research, including reviews, case reports, editorials, and conference abstracts with insufficient data.

Articles published in languages other than English.

### 2.4. Data Extraction and Management

Studies were independently screened by two reviewers based on titles and abstracts, followed by full-text evaluation. Disagreements were resolved through discussion or consultation with a third reviewer. For each included study, the following information was extracted using a standardized form: (1) Study Characteristics: Author[s], year of publication, country, study design, sample size, and follow-up duration. (2) Population Details: Mean age [±SD], breast cancer stage, and chemotherapy regimen [including cardioprotective interventions, if applicable]. (3) Biomarker Data: Type of biomarker(s), measurement time points, assessment methods, and key findings regarding their association with subsequent cardiac outcomes and reported predictive performance. (4) Outcomes: Definition of cardiotoxicity, LVEF data, survival analysis, and overall study results.

The characteristics of the included studies are summarized in Table 1.

### 2.5. Risk of Bias Assessment

The methodological quality and risk of bias were independently assessed by two researchers. For observational prognostic studies, the Quality in Prognosis Studies (QUIPS) tool was utilized, evaluating six domains: study participation, study attrition, prognostic factor measurement, outcome measurement, study confounding, and statistical analysis/reporting.

For randomized controlled trials, the Cochrane Risk of Bias 2 (RoB 2) tool was applied to assess bias arising from the randomization process, deviations from intended interventions, missing outcome data, measurement of the outcome, and selection of the reported result. Any disagreements between reviewers during the assessment process were resolved by discussion.

### 2.6. Statistical Analysis

A quantitative meta-analysis was conducted to evaluate the association between elevated troponin levels and chemotherapy-induced cardiotoxicity. For each study, 2 × 2 contingency data were extracted according to troponin status (positive vs. negative) and cardiotoxicity outcome (presence vs. absence of cardiac events or left ventricular dysfunction). The following variables were extracted: number of patients with elevated troponin who developed cardiotoxicity, number of patients with elevated troponin without cardiotoxicity, number of patients with normal troponin who developed cardiotoxicity, and number of patients with normal troponin without cardiotoxicity.

The pooled effect size was calculated as odds ratios (Ors) with 95% confidence intervals (Cis). A random-effects model using restricted maximum likelihood (REML) estimation was applied to account for between-study variability. Statistical heterogeneity among studies was assessed using Cochran’s Q test and the I^2^ statistic.

Potential publication bias was assessed through visual inspection of funnel plot asymmetry. Because fewer than ten studies were included, the results of publication bias tests were interpreted with caution.

A leave-one-out sensitivity analysis was performed by sequentially excluding each study to assess the robustness of the pooled effect estimate and to identify potential influential studies.

All statistical analyses were conducted using Stata/MP version 18.0 (StataCorp LLC, College Station, TX, USA).

## 3. Results

The database search yielded 1126 records, of which 929 remained after duplicate removal and 241 after title and abstract review. Sixty-five articles were selected for full-text evaluation. Ultimately, 14 studies met the inclusion criteria and were included in the qualitative analysis. Five studies provided sufficient data for quantitative meta-analysis.

**Figure 1:**
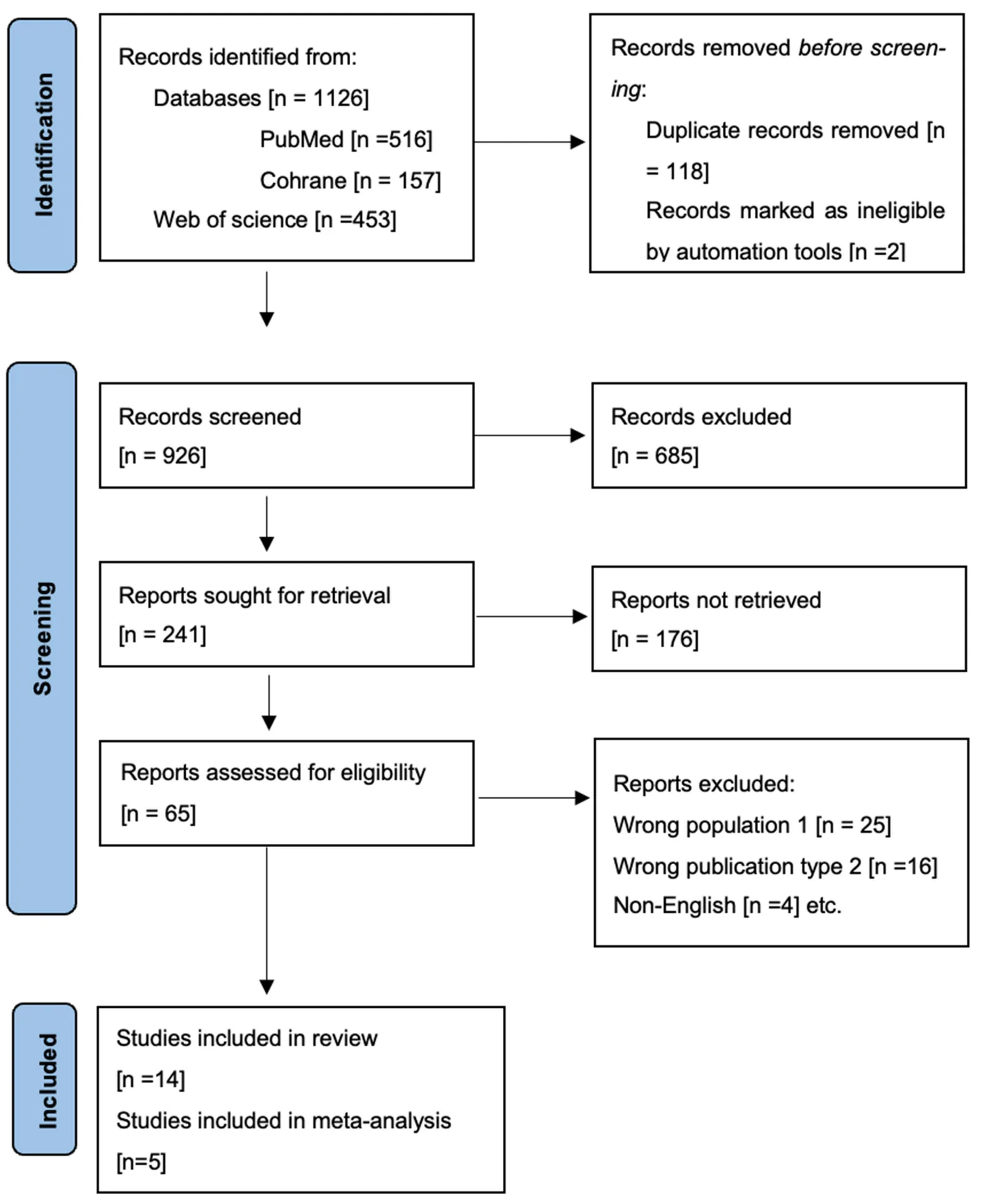
PRISMA 2020 flow diagram.

The initial search identified a total of 1126 relevant records, and after screening titles and abstracts and removing duplicates, 65 full-text articles were assessed. After meeting the eligibility criteria, 14 studies were selected for analysis. Five studies provided sufficient data for quantitative pooling in a meta-analysis of troponin elevation and chemotherapy-induced cardiotoxicity. The study selection process is presented in Figure 1.

The study included 14 papers published between 2020 and 2025 and conducted in various regions, including Europe, Asia, the Middle East, and the United States. Most studies used prospective observational cohort designs, although retrospective cohort studies, pilot studies, one randomized clinical trial, and one retrospective biomarker analysis were also presented in Table 1 [22,23,24,25,26,27,28,29,30,31,32,33,34,35].

The methodological quality of the included studies was variable (Table 2 and Table 3). Most studies were assessed as having a moderate risk of bias, primarily due to small sample size, heterogeneity in the timing of biomarker and cutoff value application, limited adjustment for confounders, and variability in cardiotoxicity definitions [22,23,24,25,28]. Some studies were assessed as having moderate-to-high or high risk of bias, particularly those with a retrospective design, included mixed populations, or contained bias in defining biomarker-based outcomes [26,27,29,30,31,32,33,34]. The only randomized trial included in the review was assessed as having low risk of bias [35]. One large prospective cohort study demonstrated a low to moderate risk of bias (Cardinale et al., 2004 [36]), whereas smaller or feasibility studies showed moderate to high risk of bias, primarily due to limited adjustment for confounders and incomplete follow-up (Sawaya et al. (2011) [37], Ky et al. (2014) [38], Feola et al. (2011) [39], Morris et al. (2011) [40]).

### 3.1. Biomarkers Investigated

Cardiac markers have been evaluated for the early detection of chemotherapy-induced cardiotoxicity. The most frequently studied biomarkers were high-sensitivity cardiac troponin (hs-cTnT) and NT-proBNP. Smaller studies have also examined additional biomarkers, such as ST2, galectin-3, inflammatory markers, and lipid-related molecules.

Troponin as an Early Biomarker. Troponin was the most frequently studied biomarker and demonstrated the most consistent association with cardiotoxicity across studies, although results varied depending on treatment setting and assay interpretation. In patients with early HER2-positive breast cancer treated with anthracyclines followed by anti-HER2 therapy, high-sensitivity troponin I (hs-TnI) levels increased significantly over time, and a sustained increase independently predicted cardiotoxicity after anthracycline exposure and during anti-HER2 therapy (Ahmed et al., 2025 [22]). In this cohort, cardiotoxicity was observed in 25% of patients, and the combination of elevated high-sensitivity C-reactive protein (hs-CRP) levels after anthracyclines and elevated hs-TnI levels during anti-HER2 therapy identified a subgroup at particularly high risk.

Similarly, in a prospective study of patients receiving trastuzumab, baseline high-sensitivity troponin T (hs-TnT) levels were significantly higher in those who subsequently developed cardiotoxicity, and patients with elevated baseline hs-TnT levels had a significantly higher event rate than those with normal baseline values (40% vs. 5%) (Canale et al., 2023 [25]). These data support the concept that elevated troponin levels may reflect preexisting subclinical myocardial injury, particularly after prior exposure to anthracyclines.

Sequential troponin elevation was also common in the anthracycline-treated patient groups. In a prospective cohort study, Dean et al. (2023) [26] found that in 41 patients receiving doxorubicin, cTnT and GDF-15 levels increased significantly after anthracycline exposure, while LVEF and LVGLS worsened during the follow-up period. However, the study was limited by a small sample size, mixed cancer types, and the lack of a clearly defined cardiotoxicity endpoint. Isemede et al. (2022) [27] also noted a progressive increase in high-sensitivity troponin T (hs-cTnT) levels during epirubicin-based chemotherapy. Researchers evaluated 81 women with breast cancer receiving epirubicin-based chemotherapy and found that hs-cTnT levels increased significantly from baseline to mid- and late-cycle (2.0 (2.0–4.7) vs. 7.4 (5.6–9.3) vs. 13.3 (8.2–22.1) ng/L; both *p* < 0.001), while NT-proBNP and sST2 showed no significant changes over time, suggesting that hs-cTnT may be a more sensitive early biomarker of anthracycline-associated myocardial injury than NT-proBNP or sST2. These data suggest that troponin is sensitive to early cardiomyocyte injury during anthracycline exposure, even before overt clinical dysfunction becomes evident.

Kozhukhov and Dovganich (2025) [29] reported that a relative increase in hsTnT of more than 81% from baseline predicted subsequent LVEF decline over 6 months (AUC 0.78, 95% CI 0.67–0.90), even when absolute values remained below the generally accepted upper reference limit. This suggests that dynamic changes in troponin can be clinically informative, even in the absence of clearly abnormal absolute concentrations.

In a large retrospective cohort study, Ju et al. (2025) [28] reported that abnormal troponin I levels were an independent predictor of cardiotoxicity, with high discriminative power (AUC = 0.871) and a multivariable odds ratio of 7.96. Zhang et al. (2022) [34] also found that hs-TnI demonstrated moderate predictive value for cardiotoxicity during anti-HER2 therapy (AUC = 0.724). However, the study emphasized that traditional clinical risk factors such as age, coronary artery disease, radiation therapy, and prior anthracycline exposure also significantly contributed to the risk.

Not all troponin test results were equally positive. Bhagat et al. (2023) [24] reported that all hs-cTnI values remained below the lower limit of detection, making troponin uninformative in this cohort. Similarly, Posch et al. (2022) [33] found that baseline hs-cTnT levels did not demonstrate convincing independent prognostic value in patients with HER2-positive early breast cancer receiving targeted therapy, although point estimates suggested a possible signal at higher cutoff values. Mecinaj et al. (2024) [30] in their retrospective analysis of 118 women from the PRADA study reported a high incidence of mild, asymptomatic cardiac dysfunction.

#### NT-proBNP as an Early Biomarker

Data on NT-proBNP/BNP were more heterogeneous. In patients receiving trastuzumab, Andersson et al. (2021) [23] found that both absolute NT-proBNP values and the change in NT-proBNP from baseline were highly predictive of trastuzumab-induced cardiotoxicity, with a cutoff value of 276.5 pg/mL or an increase of 75.8 pg/mL providing 100% sensitivity and 95% specificity. This study strongly supports the role of NT-proBNP in trastuzumab-related monitoring, although the authors noted that prior anthracycline exposure may have influenced baseline levels.

Bhagat et al. (2023) [24] also found that baseline and posttreatment NT-proBNP values correlated with subsequent LVEF decline, making NT-proBNP the strongest biomarker predictor of LVEF response in their cohort. Furthermore, Mohammed et al. (2025) [32] reported that in their retrospective analysis of stored serum/plasma samples from a previous perspective cohort study, several serum and plasma sphingolipids, specifically S1P, dhS1P, and ceramides, were associated with subsequent worsening of GLS and/or LVEF. Baseline NT-proBNP levels correlated with subsequent worsening of GLS, but NT-proBNP levels during treatment did not predict subsequent dysfunction in this cohort. Because the study focused on exploratory sphingolipid biomarkers rather than troponin or NT-proBNP as the primary prognostic factor, they were not included in the main quantitative analysis.

In contrast, several studies have found limited efficacy of natriuretic peptides. Dean et al. (2023) [26] observed no significant changes in NT-proBNP levels over time during anthracycline treatment. Isemede et al. (2022) [27] found that NT-proBNP levels did not change significantly during epirubicin-based therapy. Mohammadi et al. (2022) [31] reported stable NT-proBNP levels over 9 months and no significant deterioration in echocardiographic parameters, suggesting that NT-proBNP was not useful for early detection in a relatively low-risk group of patients receiving low-dose anthracyclines. Similarly, Mecinaj et al. (2024) [30] found that no NT-proBNP value exceeded the guideline cutoff, and NT-proBNP had little impact on the diagnosis of CTRCD in this population. A study by Posch et al. (2022) [33] also found no convincing independent association between baseline NT-proBNP levels and subsequent cardiotoxicity. In contrast to observational studies focusing on prognostic discrimination, Xia et al. (2025) [35] demonstrated that elevated NT-proBNP levels could be used in practice to trigger early cardioprotective treatment.

Taken together, NT-proBNP appears to provide information that is complementary rather than equivalent to troponin. Whereas troponin primarily reflects myocardial injury, NT-proBNP reflects myocardial wall stress and hemodynamic load. Its association with subsequent cardiac dysfunction was less consistent across the included studies and appeared to depend on treatment context, timing of measurement, and patient risk profile.

### 3.2. Other Emerging Biomarkers

The most frequently studied biomarkers were hs-cTnT and NT-proBNP. In addition, several studies have investigated new biomarkers, including soluble ST2, galectin-3, inflammatory markers, and lipid-related molecules, although these were evaluated in smaller patient groups (Bhagat et al., 2023 [24]; Isemede et al., 2022 [27]; Dean et al., 2023 [26]; Mohammed et al., 2025 [32]). These biomarkers have shown potential associations with myocardial injury or oxidative stress during chemotherapy. It is worth noting that the available data remain limited and heterogeneous. Ahmed et al. (2025) [22] found that high-sensitivity C-reactive protein (hs-CRP) independently predicted cardiotoxicity at baseline, after anthracycline treatment, and after anti-HER2 therapy, and that the interaction between inflammatory activation and elevated troponin levels provided particularly strong prognostic information. IL-6 and uric acid had no prognostic significance in this study.

Bhagat et al. (2023) [24] reported that changes in ST2 and galectin-3 were associated with subsequent decline in LVEF, while galectin-3 also correlated with changes in left ventricular hypertrophy. Dean et al. (2023) [26] found that GDF-15 levels increased significantly during anthracycline treatment, and that galectin-3 correlated with changes in LVEF, although neither biomarker correlated significantly with LVGLS. Mohammed et al. (2025) [32] further identified several baseline and treatment-related sphingolipid species associated with subsequent deterioration in LVEF or GLS, suggesting that lipidomic biomarkers may become increasingly valuable prognostic factors.

Overall, these unconventional biomarkers remain promising but are still in the exploratory stage as supporting data are limited by small sample sizes, methodological heterogeneity, and lack of replication.

### 3.3. Cardiac Function Outcomes

Most studies assessed cardiotoxicity by measuring changes in LVEF using echocardiography. Biomarker increases often preceded measurable LVEF declines, suggesting that circulating biomarkers may provide an earlier signal of myocardial injury than imaging alone. The reported incidence of cardiotoxicity varied significantly across studies, ranging from 4.4% in a cohort of patients receiving trastuzumab (Andersson et al., 2021) [23] to approximately 25% in a prospective cohort of HER2-positive patients (Ahmed et al., 2025) [22] and 18.1% overall in a large retrospective study (Ju et al., 2025 [28]). The study by Zhang et al. (2022) [34] observed cardiotoxicity in 15.9% of patients during anti-HER2 therapy, with most cases representing asymptomatic decreases in LVEF. Posch et al. (2022) [33] reported cardiotoxicity in 10% of patients, all cases occurring during HER2-targeted therapy, while Bhagat et al. (2023) [24] found frequent subclinical cardiotoxicity, including dysfunction measured by GLS, in 27% of patients’ post-therapy and 38% at 6 months.

Where a decrease in LVEF was reported, it often followed rather than preceded a rise in biomarkers. Ahmed et al. (2025) [22] observed that mean LVEF in the cardiotoxicity group decreased from 68% at baseline to 49%, while Bhagat et al. (2023) [24] found that changes in NT-proBNP, ST2, and galectin-3 correlated with subsequent LVEF response. In contrast, some studies suggest that imaging remains more effective than biomarkers for dynamic risk prediction; for example, Posch et al. (2022) [33] found that baseline and longitudinal LVEF dynamics were superior to hs-cTnT and NT-proBNP in predicting trastuzumab-associated cardiotoxicity.

A study by Mecinaj et al. (2024) [30] reported a cardiotoxicity incidence rate exceeding 50% when defined using biomarker-based criteria, although most cases were mild and asymptomatic.

### 3.4. Time of Biomarker Measurement

Across studies, biomarkers were measured at various time points, including baseline, during chemotherapy cycles, and after completion of treatment. Early increases in biomarker levels during treatment cycles were often associated with subsequent cardiac dysfunction, highlighting the potential value of serial biomarker monitoring. For example, Ahmed et al. (2025) [22] found that hs-TnI after anti-HER2 therapy and hs-CRP after anthracyclines had stronger prognostic value than baseline values alone, while Kozhukhov and Dovganich (2025) [29] demonstrated that the percentage increase in hsTnT from baseline had prognostic value even within the normal reference range. Similarly, Andersson et al. (2021) [23] found that changes in NT-proBNP levels were highly predictive of trastuzumab-induced cardiotoxicity. NT-proBNP levels in the control group decreased after 6 months.

These findings suggest that serial biomarker assessment may provide more information than isolated baseline measurements. However, the optimal timing and frequency of measurement remain uncertain.

### 3.5. Cardioprotective Interventions

Only a small number of included studies directly addressed cardioprotective interventions. Bhagat et al. (2023) [24] reported that initiating or changing cardioprotective medications such as beta-blockers, ACE inhibitors, or angiotensin II receptor blockers did not significantly impact changes in LVEF or GLS in their small pilot cohort. Mecinaj et al. (2024) [30], in a retrospective analysis of biomarkers conducted as part of a randomized trial of metoprolol and candesartan, found that these medications did not reduce the incidence of biomarker-defined cardiac dysfunction associated with CTRC. In contrast, Xia et al. (2025) [35] demonstrated that a cardioprotective strategy based on NT-proBNP levels is feasible, safe, and clinically relevant, with increases in NT-proBNP levels occurring early and prompting rapid initiation or titration of neurohormonal therapy.

### 3.6. Summary of Findings

Overall, the included studies reported associations between early changes in cardiac biomarkers, particularly high-sensitivity cardiac troponin and NT-proBNP, and subsequent myocardial injury or cardiac dysfunction (Table 4). However, the magnitude and consistency of these associations varied across studies. Significant heterogeneity in study design, biomarker assays and cutoff values, timing of measurement, treatment regimens, and definitions of cardiac outcomes limits direct comparability across studies. Also, it precludes firm conclusions regarding the predictive performance of these biomarkers.

### 3.7. Meta-Analysis of the Association Between Troponin Elevation and Cardiotoxicity

Five studies were included (Cardinale et al., 2004 [36]; Sawaya et al., 2011 [37]; Ky et al., 2014 [38]; Feola et al., 2011 [39]; Morris et al., 2011 [40]) in the quantitative meta-analysis evaluating the prognostic value of troponin for chemotherapy-induced cardiotoxicity. Across the included studies, the direction and magnitude of the association between elevated troponin levels and subsequent cardiac outcomes varied considerably.

**Figure 2:**
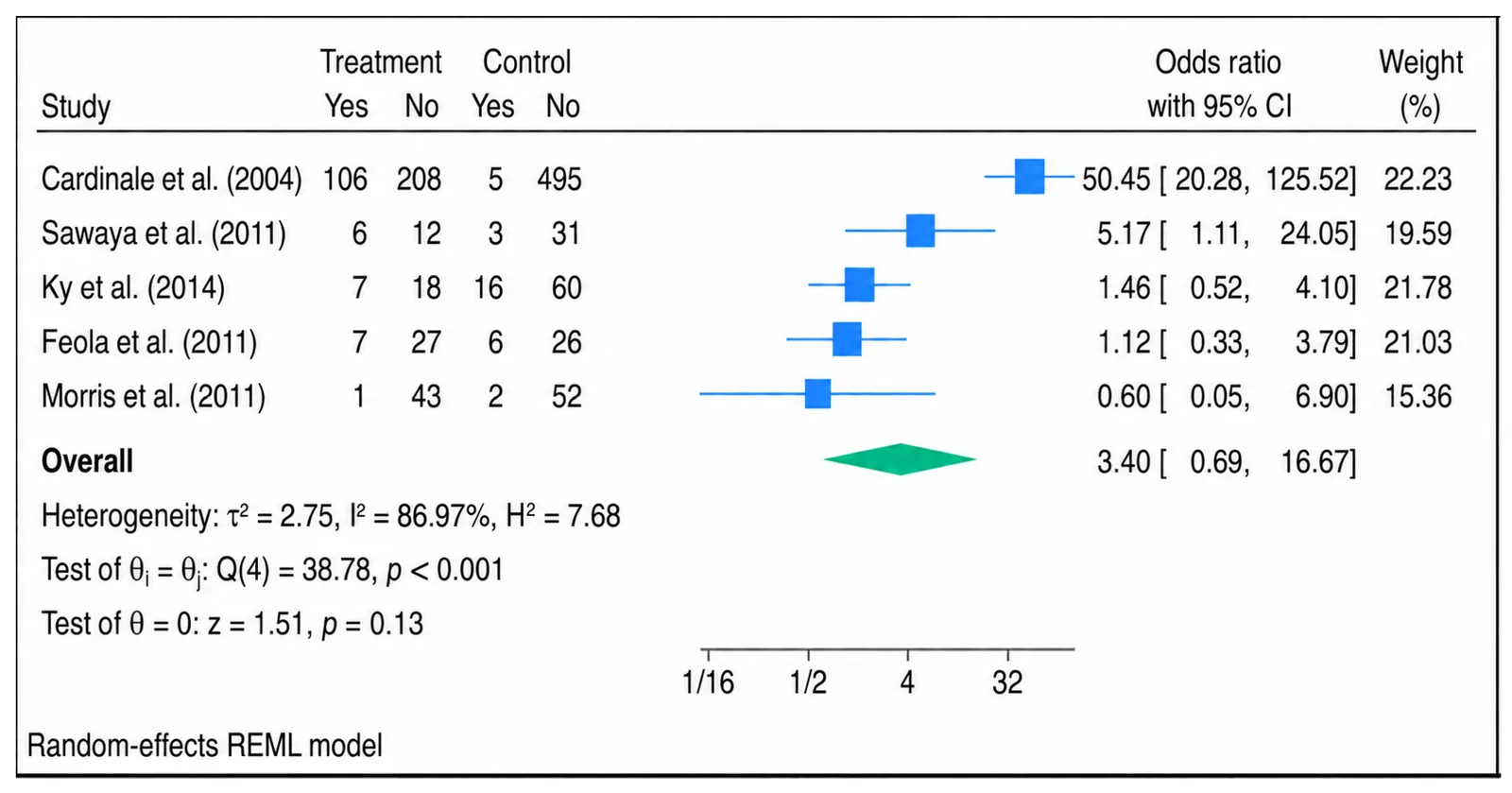
Forest plot of the association between elevated troponin levels and chemotherapy-induced cardiotoxicity. Included studies were Cardinale et al. (2004) [36], Sawaya et al. (2011) [37], Ky et al. (2014) [38], Feola et al. (2011) [39], and Morris et al. (2011) [40]. The pooled odds ratio was calculated using a random-effects REML model.

The pooled analysis using a random-effects REML model demonstrated that patients with elevated troponin had higher odds of developing cardiotoxicity compared with those with normal troponin levels; however, this association did not reach statistical significance [OR = 3.40, 95% CI 0.69–16.67, *p* = 0.13] (Figure 2).

Substantial heterogeneity was observed across studies [τ^2^ = 2.75, I^2^ = 86.97%], indicating considerable variability in effect estimates. Cochran’s Q test also indicated significant heterogeneity [Q = 38.78, *p* < 0.001]. This variability may be explained by differences in study populations, chemotherapy regimens, troponin measurement protocols, and definitions of cardiotoxicity.

Sensitivity analysis (leave-one-out). A leave-one-out sensitivity analysis was conducted to evaluate the influence of individual studies on the pooled effect estimate.

Exclusion of Cardinale et al. (2004) [36] resulted in a complete elimination of between-study heterogeneity [I^2^ = 0%, τ^2^ = 0], indicating that this study was the primary source of heterogeneity in the meta-analysis. In addition, the pooled effect size was substantially attenuated and remained statistically non-significant following its exclusion. The apparent association between troponin elevation and subsequent cardiac dysfunction should therefore be interpreted cautiously, as the pooled estimate was substantially influenced by a single study with an extreme effect size.

In contrast, removal of any of the other included studies did not materially alter the pooled estimates or heterogeneity, with I^2^ values remaining above 85% in all cases. These findings suggest that the overall results are strongly influenced by a single study reporting an extreme effect size.

These findings highlight the importance of interpreting the pooled estimate with caution, as the observed association may be driven by a single influential study rather than a consistent effect across the evidence base.

### 3.8. Publication Bias

**Figure 3:**
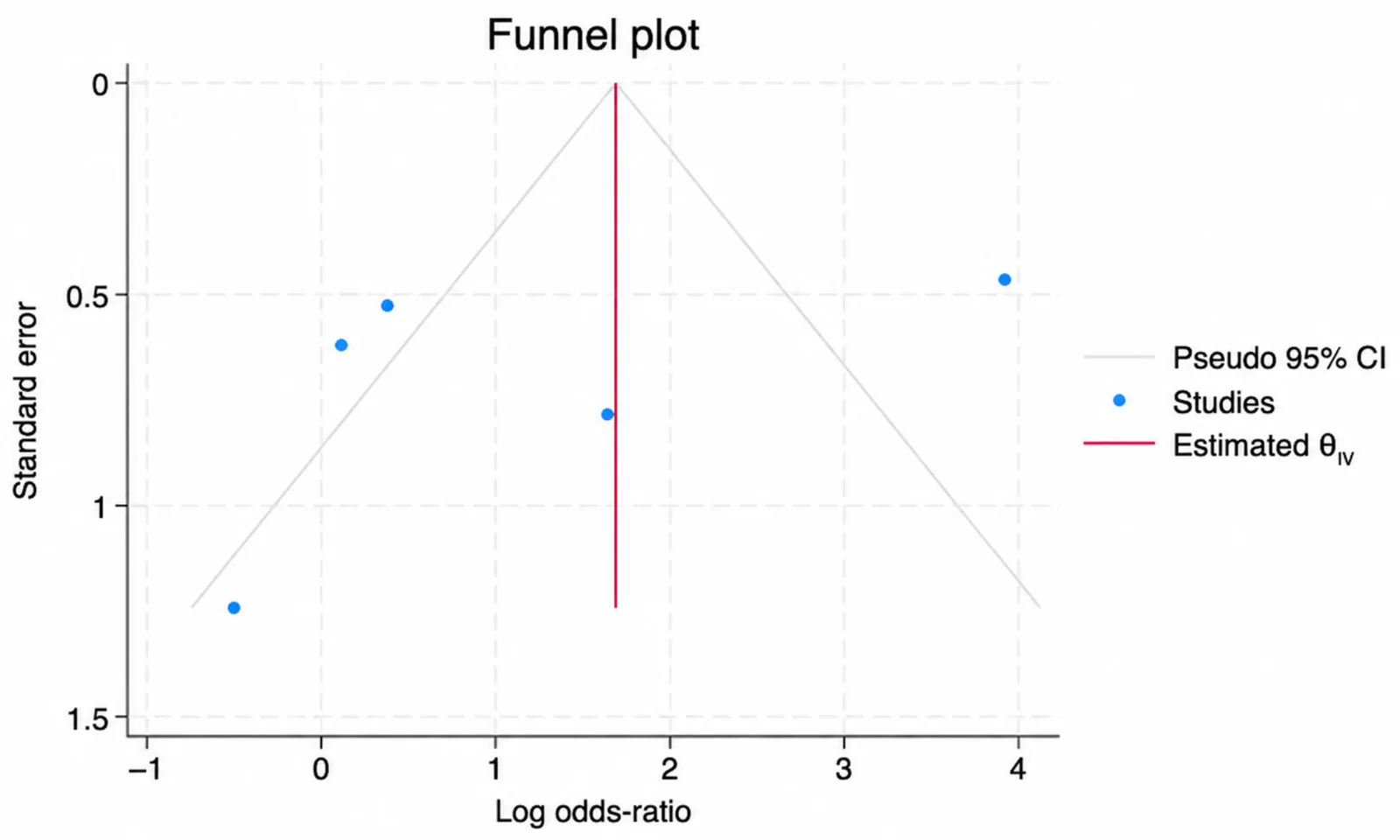
Funnel plot assessing potential publication bias among the included studies.

Visual inspection of the funnel plot suggested some degree of asymmetry (Figure 3). However, interpretation of this finding is limited due to the small number of included studies (*n* = 5), which reduces the reliability of the funnel plot assessment.

In addition, the observed asymmetry is likely influenced by substantial between-study heterogeneity and the presence of an influential study with an extreme effect estimate, rather than true publication bias.

Therefore, no firm conclusions regarding publication bias can be drawn. Egger’s regression test was not considered reliable due to the limited number of studies.

## 4. Discussion

This systematic review and meta-analysis assessed the role of early cardiac markers in detecting chemotherapy-induced cardiotoxicity. In the quantitative synthesis, elevated troponin levels were associated with higher odds of cardiotoxicity; however, this association did not reach statistical significance [OR = 3.40, 95% CI 0.69–16.67]. Importantly, substantial between-study heterogeneity was observed [I^2^ = 86.97%], indicating considerable variability in effect estimates across studies. Sensitivity analysis further demonstrated that the pooled estimate was strongly influenced by a single study (Cardinale et al., 2004 [36]), which reported an extreme effect size. Exclusion of this study resulted in complete resolution of heterogeneity [I^2^ = 0%] and attenuation of the pooled effect estimate, suggesting that the overall association may be driven by study-level differences rather than a consistent effect across the evidence base. These findings highlight that, although troponin elevation is biologically plausible and consistently associated with myocardial injury, the strength and consistency of its predictive value for clinically relevant cardiotoxicity remain uncertain. Variability in chemotherapy regimens, timing of biomarker assessment, troponin assay sensitivity, and definitions of cardiotoxicity likely contribute to the observed heterogeneity. Overall, multiple studies have shown that elevated troponin levels during chemotherapy are associated with a subsequent reduction in LVEF and an increased risk of cardiotoxic events (Ahmed et al., 2025 [22]; Canale et al., 2023 [25]; Ju et al., 2025 [28]). Taken together, the available evidence supports the role of troponin as an early marker of myocardial injury, although its prognostic utility requires further validation in larger and more standardized studies.

These findings emphasize the need to distinguish biological injury from subsequent functional cardiac impairment. Troponin primarily reflects cardiomyocyte injury, whereas NT-proBNP reflects myocardial wall stress and hemodynamic load. In contrast, GLS and LVEF characterize functional consequences of evolving cardiac injury. Biomarker elevation should not be interpreted as equivalent to CTRCD. Rather, circulating biomarkers may provide early biological signals that complement, but do not replace, cardiac imaging and clinical assessment.

Dynamic changes in troponin levels may be more informative than absolute cutoff values. Even a relative increase within the normal reference range predicted subsequent cardiac dysfunction (Kozhukhov and Dovganych, 2025 [29]). In contrast, NT-proBNP demonstrated inconsistent prognostic performance. Some studies reported strong associations, particularly in populations treated with trastuzumab (Andersson et al., 2021) [23], while others found limited or no predictive value (Dean et al., 2023 [26]; Mohammadi et al., 2022 [31]; Posch et al., 2022 [33]). This variability likely reflects different pathophysiological processes recorded by NT-proBNP, which primarily reflect hemodynamic stress rather than direct myocardial injury. Novel biomarkers, including ST2, galectin-3, GDF-15, and sphingolipids, have shown potential associations with early cardiac dysfunction (Bhagat et al., 2023 [24]; Dean et al., 2023 [26]; Mohammed et al., 2025 [32]). However, available data remain preliminary and insufficient for routine clinical use. The results of the study by Mecinaj et al. (2024) [30] suggest that biomarker-defined cardiotoxicity may not always predict long-term dysfunction, highlighting the complexity of biomarker interpretation and the potential impact of study design and outcome definitions.

The results of this review are generally consistent with previous studies, showing that cardiac troponin is one of the most informative early biomarkers of chemotherapy-associated myocardial injury, particularly in patients receiving anthracyclines and subsequent anti-HER2 therapy. These findings are also supported by previous meta-analytic evidence. A comprehensive meta-analysis by Michel et al. (2020) [41] demonstrated that both cardiac troponins and natriuretic peptides are significantly associated with an increased risk of cancer therapy-related cardiotoxicity across diverse cancer populations, highlighting their role as early indicators of myocardial injury. However, unlike Michel et al., which included heterogeneous cancer types and treatment regimens, the present analysis focused specifically on breast cancer patients receiving anthracycline- and HER2-targeted therapies, which may partly explain the variability in effect estimates observed in this study. In a prospective cohort study of breast cancer, Sawaya et al. (2012) [42] found that early changes in ultrasensitive troponin I, particularly in conjunction with myocardial strain, were associated with subsequent cardiotoxicity, supporting the concept that biochemical injury can be detected before overt deterioration of normal left ventricular function.

Additional longitudinal data confirmed the importance of serial biomarker assessment rather than isolated baseline measurements. In a multicenter study of women receiving doxorubicin, taxanes, and trastuzumab, Putt et al. (2015) [43] found that longitudinal changes in several biomarkers were associated with cardiotoxicity over the course of treatment. These findings emphasize that time-dependent biomarker trajectories may better reflect evolving myocardial injury than single-threshold approaches. More recent observational studies have also shown that biomarker behavior can vary depending on the treatment context. In patients receiving therapy for advanced breast cancer, Demissei et al. (2020) [44] reported that longitudinal changes in cardiovascular biomarkers were associated with CTRC, with stronger signals observed in patients receiving sequential anthracycline and trastuzumab therapy. These results are consistent with the present review, which found that troponin was most informative in higher-risk settings, while NT-proBNP showed more variable efficacy across studies. In the GeparOcto-GBG 84 study, Rüger et al. (2020) [45] found that NT-proBNP was associated with cardiotoxic reactions in patients with early-stage breast cancer receiving intensive and escalated chemotherapy regimens, while high-sensitivity troponin T was not significantly associated in this cohort. This supports the idea that the prognostic value of natriuretic peptides may depend on the intensity of the treatment regimen, timing of sampling, and the patient’s risk profile.

These results are also consistent with broader recommendations in cardio-oncology. The 2022 European Society of Cardiology guidelines (Lyon et al., 2022) [5] for cardio-oncology recommend considering cardiac troponin and natriuretic peptides for basic risk stratification and treatment monitoring in selected patients receiving potentially cardiotoxic therapy, while acknowledging that the evidence base is still limited and that biomarker interpretation should be integrated with imaging data and the clinical context.

### 4.1. Clinical Implications

Cardiac troponin and NT-proBNP are clinically relevant components of cardio-oncology surveillance. They are incorporated into current guideline-based monitoring strategies for selected patients receiving potentially cardiotoxic cancer therapy [5]. The findings of this review suggest that cardiac biomarkers may provide complementary information during cardio-oncology surveillance when interpreted alongside cardiac imaging, clinical risk factors, and treatment characteristics. Serial measurement of troponin and NT-proBNP may help identify early biological changes during potentially cardiotoxic therapy. However, the current evidence does not establish these biomarkers as standalone predictors of CTRCD. Early identification provides an opportunity to implement preventive and therapeutic strategies, including intensified cardiac monitoring, modification of chemotherapy regimens, and initiation of cardioprotective therapy (e.g., ACE inhibitors, beta-blockers). Further prospective studies are needed to determine whether biomarker-guided monitoring and interventions improve clinically meaningful cardiovascular outcomes.

A randomized trial by Xia et al. (2025) [35] demonstrated that NT-proBNP-guided patient management resulted in early initiation of cardioprotective therapy and improved short-term cardiac outcomes, suggesting the potential value of biomarker-guided strategies in cardio-oncology.

### 4.2. Explanation of Heterogeneity

The included studies demonstrated significant heterogeneity, particularly in the meta-analysis [I^2^ = 86.97%], indicating significant variability in effect estimates. The studies enrolled patients receiving anthracyclines, trastuzumab, or combination therapy. Each therapy is associated with different mechanisms and timing of cardiotoxicity. Secondly, differences in troponin assay methods, including the use of conventional and highly sensitive assays, as well as different cutoff values, may have impacted the detection and interpretation of biomarker elevations. Furthermore, heterogeneity in defining cardiotoxicity across studies included variations in LVEF decline, GLS changes, and composite clinical endpoints. The timing of biomarker measurements also varied significantly across studies. Assessments were performed at baseline, during treatment, or after completion of treatment, which may impact the detection of early and late myocardial injury. Finally, differences were identified in the duration of follow-up, ranging from short-term monitoring to long-term outcome assessment. Taken together, these differences limit direct comparability across studies and highlight the need for standardization of definitions and protocols in future studies.

### 4.3. Strengths

This study has several important strengths. First, the review was conducted in accordance with PRISMA guidelines and pre-registered in PROSPERO. This reduced the risk of bias in reporting results and increased methodological transparency. Second, both a qualitative synthesis and a quantitative meta-analysis were conducted, allowing for a comprehensive assessment of the available data. Third, the inclusion of multiple cardiac markers in various chemotherapy regimens provides a broad and clinically relevant overview of biomarker efficacy in various treatment contexts.

### 4.4. Limitations

Several limitations should be noted. The meta-analysis included a relatively small number of studies, which may limit the reliability and generalizability of the pooled estimates. Significant heterogeneity was observed between studies. The pooled troponin estimate was highly sensitive to a single influential study. Exclusion of Cardinale et al. (2004) [36] eliminated between-study heterogeneity and attenuated the pooled effect, indicating limited robustness of the overall estimate. Therefore, the pooled OR should not be interpreted as establishing a consistent predictive relationship between troponin elevation and subsequent CTRCD. Variability in the timing of biomarker measurements further complicates the interpretation of the results. Furthermore, most of the included studies were observational, limiting the ability to establish causal relationships and creating the potential for residual confounding. These factors should be considered when interpreting the results of this review.

Further research should focus on identifying the optimal timing and frequency of biomarker measurements, as well as their clinically relevant cutoff values for different patient groups and treatment regimens. Randomized trials evaluating biomarker-based cardioprotective strategies are also needed to determine whether early intervention based on biomarker changes can improve long-term cardiovascular outcomes.

## 5. Conclusions

This systematic review and meta-analysis suggest that early changes in cardiac biomarkers, particularly troponin, may provide useful signals of myocardial injury during potentially cardiotoxic cancer therapy. However, the association between elevated troponin level and cancer therapy-related cardiac dysfunction did not reach the threshold of statistical significance and showed substantial heterogeneity. NT-proBNP demonstrated more variable performance and may provide complementary information regarding myocardial wall stress. Overall, circulating biomarkers should currently be considered complementary to cardiac imaging rather than established standalone predictors of CTRCD. Further large prospective studies using standardized biomarker assays, sampling time points, and CTRCD definitions are required to establish their predictive performance and clinical utility.

## 6. Clinical Perspectives

### 6.1. Competence in Medical Knowledge

Early elevations in cardiac biomarkers may provide complementary information during anthracycline and HER2-targeted therapy in patients with breast cancer. Troponin primarily reflects myocardial injury, whereas NT-proBNP reflects myocardial wall stress and hemodynamic load. Serial biomarker monitoring may help identify early biological changes before measurable deterioration in left ventricular function. However, their predictive value for subsequent CTRCD remains uncertain.

### 6.2. Competence in Patient Care

Troponin and NT-proBNP may provide complementary information when interpreted alongside cardiac imaging, clinical risk factors, and treatment characteristics. Biomarker changes may help identify patients who warrant closer cardiac surveillance. However, current evidence does not establish these biomarkers as standalone predictors of CTRCD or support biomarker-guided cardioprotective interventions in routine clinical practice.

### 6.3. Prospects for Implementation into Clinical Practice

**Figure 4:**
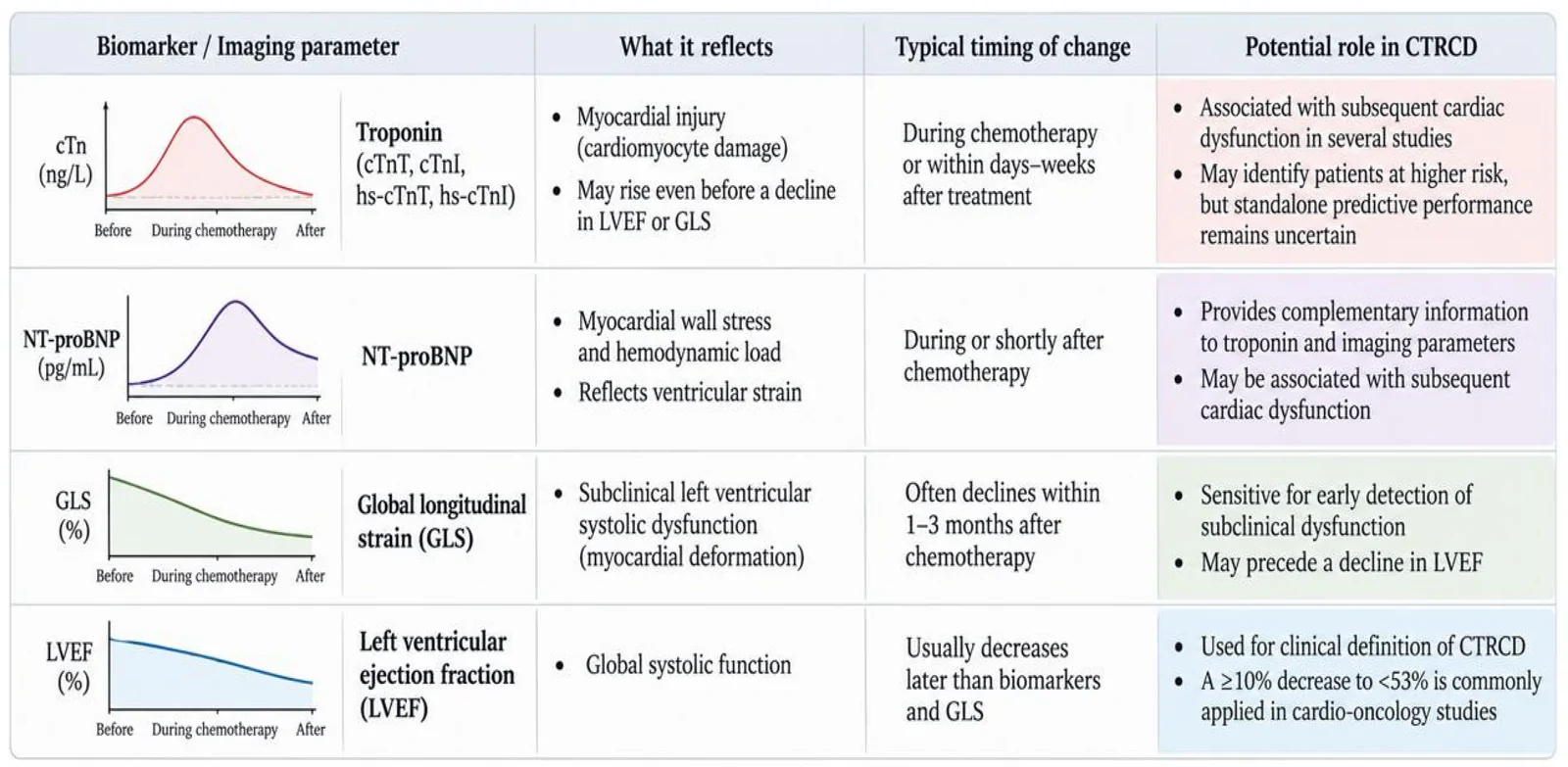
Interrelationship between cardiac biomarkers, imaging parameters, and CTRCD. Cardiac troponin reflects myocardial injury and may rise during treatment before measurable changes in cardiac function occur. NT-proBNP reflects myocardial wall stress and hemodynamic load and can provide additional information. GLS can detect subclinical left ventricular dysfunction, whereas changes in LVEF typically reflect functional impairment at later stages. The depicted trajectories are conceptual and are not intended to represent quantitative pooled estimates.

Standardization of biomarker thresholds, assay types, measurement timing and CTRCD definitions is necessary before routine clinical implementation. Future prospective randomized trials should evaluate whether biomarker-guided interventions improve long-term cardiovascular outcomes and survival in breast cancer patients receiving cardiotoxic therapy (Figure 4).

## Figures and Tables

**Table 1:** Characteristics of Included Studies.

Study [Year]	Country	Study Design	Sample Size	Population	Chemotherapy Regimen	Biomarkers Assessed	Main Outcome
Ahmed et al., 2025 [22]	Syria	Prospective cohort	44	HER2+ breast cancer	AC + trastuzumab	hs-cTnI, NT-proBNP	Early cardiotoxicity
Andersson et al., 2021 [23]	Sweden	Prospective observational cohort	136	HER2+ breast cancer	Trastuzumab	NT-proBNP	TIC ^1^
Bhagat et al., 2023 [24]	USA	Prospective pilot cohort	31	breast cancer	Anthracyclines ± trastuzumab	hs-cTnI, NT-proBNP, ST2, Gal-3	Subclinical toxicity
Canale et al., 2023 [25]	Italy	Prospective single-arm cohort [3T trial]	24	HER2+ breast cancer	Trastuzumab	hs-cTnT	Cardiotoxicity
Dean at al., 2023 [26]	USA	Prospective cohort	41	Mixed cancers incl. breast	Anthracyclines	hs-cTnT, NT-proBNP, ST2	Cardiotoxicity
Isemede et al., 2022 [27]	UK	Prospective longitudinal observational	81	Breast cancer	Epirubicin-based	hs-cTnT, NT-proBNP, ST2	Subclinical cardiotoxicity
Ju et al., 2025 [28]	China	Retrospective cohort	415	Breast cancer	Anthracyclines ± trastuzumab	hs-cTnI, BNP	Cardiotoxicity risk
Kozhukhov et al., 2025 [29]	Ukraine	Prospective cohort	60	Breast cancer	Anthracyclines ± trastuzumab	hs-cTnT	Cardiac injury prediction
Mecinaj et al., 2024 [30]	Norway	Retrospective cohort	118	Breast cancer	Epirubicin-based	hs-cTnT, NT-proBNP	CTRCD ^2^
Mohammadi et al., 2022 [31]	Iran	Prospective observational	57	Breast cancer	Low-dose anthracyclines	Low-dose anthracyclines	The absence of events
Mohammed et al., 2025 [32]	USA	Biomarker analysis cohort	20	Breast cancer	Anthracyclines ± taxanes	NT-proBNP, sphingolipids	Cardiac injury markers
Posch et al., 2022 [33]	Austria	Retrospective cohort	185	Early breast cancer	Anthracyclines	hs-cTnT, NT-proBNP	CTRCD
Zhang et al., 2022 [34]	China	Retrospective cohort	420	HER2+ breast cancer	Trastuzumab ± pertuzumab	hs-cTnI	CTRCD
Xia et al., 2025 [35]	USA	Randomized trial	100	Breast cancer	Anthracycline-based	NT-proBNP	Cardiac injury markers

^1^ TIC = trastuzumab-induced cardiotoxicity; ^2^ CTRCD = cancer therapy-related cardiac dysfunction.

**Table 2:** Risk of bias assessment of included studies using the Quality in Prognosis Studies [QUIPS].

Study [Year]	Study Participation	Study Attrition	Prognostic Factor Measurement	Outcome Measurement	Confounding	Statistical Analysis	Overall RoB
Cardinale et al., 2004 [36]	Low	Moderate	Low	Low	Moderate	Moderate	Moderate
Sawaya et al., 2011 [37]	Low	Moderate	Low	Low	Moderate	Moderate	Moderate
Ky et al., 2014 [38]	Low	Moderate	Moderate	Low	Moderate	Moderate	Moderate
Feola et al., 2011 [39]	Moderate	Moderate	Moderate	Low	High	Moderate	High
Morris et al., 2011 [40]	Moderate	High	Moderate	Low	High	High	High
Andersson et al., 2021 [23]	Low	Low	Low	Low	Moderate	Low	Low
Mohammadi et al., 2022 [31]	Moderate	Moderate	Moderate	Moderate	High	Moderate	High
Isemede et al., 2022 [27]	Low	Low	Low	Low	Moderate	Low	Low
Zhang et al., 2022 [34]	Moderate	Moderate	Moderate	Moderate	High	Moderate	High
Posch et al., 2022 [33]	Low	Low	Low	Low	Moderate	Low	Low
Canale et al., 2023 [25]	Moderate	Low	Low	Moderate	Moderate	Moderate	Moderate
Bhagat et al., 2023 [24]	Moderate	Moderate	Moderate	Low	High	Moderate	High
Dean et al., 2023 [26]	Moderate	Moderate	Moderate	Low	High	Moderate	High
Mecinaj et al., 2024 [30]	Low	Low	Low	Low	Moderate	Low	Low
Ahmed et al., 2025 [22]	Moderate	Low	Low	Low	Moderate	Moderate	Moderate
Kozhukhov et al., 2025 [29]	Moderate	Low	Low	Moderate	Moderate	Moderate	Moderate
Ju et al., 2025 [28]	Moderate	Moderate	Moderate	Moderate	High	Moderate	High
Mohammed et al., 2025 [32]	Moderate	High	Moderate	Moderate	High	Moderate	High

**Table 3:** Risk of Bias Assessment [RoB 2–RCTs].

Study	Randomization Process	Deviations from Intended Interventions	Missing Outcome Data	Outcome Measurement	Selection of Reported Results	Overall Risk
Xia et al., 2025 [35]	Low	Low	Low	Low	Moderate	Low

**Table 4:** Summary of the association between early cardiac markers and chemotherapy-induced cardiotoxicity in patients with breast cancer.

Biomarker	No. of Studies	Direction of Association	Key Findings	Consistency	Strength of Evidence
High-sensitivity cardiac troponin [hs-cTnI/hs-cTnT]	High [majority of studies]	Generally positive	Early troponin elevation was generally associated with subsequent myocardial injury or decline in cardiac function; however, the magnitude and statistical significance of this association varied considerably between studies.	Variable	Moderate; limited by substantial heterogeneity
NT-proBNP/BNP	Moderate–High	Variable association	NT-proBNP elevations were associated with subsequent cardiac dysfunction in some studies, whereas others reported limited or no prognostic value; findings may reflect myocardial wall stress rather than direct myocardial injury.	Moderate to low	Moderate
Combined biomarkers [troponin + NT-proBNP]	Several	Potentially complementary association	Combined assessment may provide complementary information for risk stratification; however, evidence for improved predictive performance compared with individual biomarkers remains limited.	Variable	Limited–Moderate
sST2	Limited	Positive trend	Elevated sST2 associated with early myocardial stress and subclinical dysfunction	Limited evidence	Low–Moderate
Galectin-3	Limited	Positive trend	Associated with fibrosis and remodeling but inconsistent predictive value	Low consistency	Low
Inflammatory markers [CRP, IL-6, TNF-α]	Few	Mixed	Elevated inflammatory markers may correlate with cardiotoxicity but not specific	Low consistency	Low
Lipid-related biomarkers [sphingolipids]	Very limited	Emerging association	Early evidence suggests association with myocardial injury pathways	Very limited evidence	Very low

## Data Availability

No new data were created or collected in this study. Data analyzed in this systematic review and meta-analysis were derived from previously published studies, which are cited in the manuscript. The data extracted and analyzed in this study are available from the corresponding author upon reasonable request.

## Supplementary Materials

The following supporting information can be downloaded at: https://www.mdpi.com/article/10.3390/jcm15207882/s1, Table S1: PRISMA 2020 Checklist. Table S2. Search strategy.

## Abbreviations

The following abbreviations are used in this manuscript:

BNP	B-type natriuretic peptide
cTn	Cardiac Troponin
CTRCD	Cancer Therapy-Related Cardiac Dysfunction
ESC	European Society of Cardiology
GLS	Global Longitudinal Strain
hs-cTnI	high-sensitivity cardiac troponin I
hs-cTnT	high-sensitivity cardiac troponin T
LVEF	left ventricular ejection fraction
NT-proBNP	N-terminal pro-B-type natriuretic peptide
PRISMA	Preferred Reporting Items for Systematic Reviews and Meta-Analyses
