# Supplementary material for: Early Cardiac Biomarkers for the Detection of Cancer Therapy-Related Cardiac Dysfunction in Breast Cancer: A Systematic Review and Meta-Analysis
Source: J Clin Med. 2026 Oct 13;15(20):7882. doi: 10.3390/jcm15207882 (PMC13706556; doi:10.3390/jcm15207882)
Supplement: Supplementary file 1 [file jcm-15-07882-s001.zip › jcm-4553685-supplementary table S2.pdf]

Supplemental Table S2. Search strategy

Pubmed	(cardiotoxicity[Title/Abstract] OR cardiomyopathy[Title/Abstract]) AND (anthracycline[Title/Abstract] OR doxorubicin[Title/Abstract])
Scopus	("breast cancer" OR "breast neoplasm*" OR "breast carcinoma") AND (cardiotoxic* OR "chemotherapy-induced cardiotoxicity" OR "cancer therapy-related cardiac dysfunction" OR CTRCD OR "cardiomyopath*") AND (troponin OR "cardiac troponin" OR hs-cTnI OR hs-cTnT OR "high sensitivity troponin" OR "NT-proBNP" OR BNP OR "natriuretic peptide*") AND (chemotherapy OR anthracycline* OR doxorubicin OR epirubicin OR trastuzumab OR "HER2-targeted therapy"))

**Web of Science Search Strategy**	TS=(cardiotoxicity OR "cardiac toxicity" OR "cardiac dysfunction"<br>OR cardiomyopathy OR "heart failure"<br>OR "left ventricular dysfunction" OR "myocardial injury"<br>)<br>AND<br>TS=(chemotherapy OR "antineoplastic agents"<br>OR "anticancer drugs" OR cytotoxic<br>)<br>AND<br>TS=(cancer OR malignancy OR neoplasm OR tumor<br>)
